# Artificial intelligence for predicting 30-day mortality after emergency laparotomy

**DOI:** 10.1007/s00068-026-03319-w

**Published:** 2026-08-31

**Authors:** Sergei Bedrikovetski, Ishraq Murshed, Warren Seow, Zachary Bunjo, Jahan Singh De Fontgalland, Luke Traeger, Ryash Vather

**Affiliations:** 1 Discipline of Surgery, School of Medicine, College of Health, Adelaide University, Adelaide, SA Australia; 2 Colorectal Unit, Department of Surgery, 5E 334, Royal Adelaide Hospital, Port Road, Adelaide, SA 5000 Australia; 3 School of Public Health, College of Health, Adelaide University, Adelaide, SA Australia

**Keywords:** Artificial intelligence, Emergency laparotomy, Mortality, National emergency laparotomy audit

## Abstract

**Purpose:**

Accurate pre-operative risk prediction in emergency laparotomy (EL) is essential for appropriate resource allocation and improve patient outcomes. This study aimed to establish the accuracy of an artificial intelligence (AI) model in predicting 30-day mortality after EL using a national dataset.

**Methods:**

Data was extracted from the Australian and New Zealand Emergency Laparotomy Audit-Quality Improvement (ANZELA-QI) database from 1 July 2018 to 24 July 2023. AI models were created employing multilayer perceptron (MLP) and radial basis function (RBF) architectures. Extended AI models were developed using all available pre-operative variables and simplified AI models were developed using statistically significant variables identified through univariate analysis. We assessed the performance of AI models with that of a multivariate logistic regression (LR) and the UK-based National Emergency Laparotomy Audit (NELA) models using the area under the receiver operator characteristic curve (AUROC).

**Results:**

Data from 8293 ELs were included in the model and were randomly divided into a training dataset of 6619 (80%) and a testing dataset of 1674 (20%). There were 537 (6.5%) deaths 30 days after EL. In the testing dataset, the NELA model performed the best (AUROC 0.836), followed by the multivariate LR model (0.824), MLP simplified (0.817). MLP extended (0.802), RBF extended (0.719), and RBF simplified (0.672). Pairwise comparisons showed no significant difference in discrimination among the NELA, multivariate LR and MLP models.

**Conclusion:**

Pre-operative MLP and multivariate LR models demonstrated comparable performance to the NELA model in predicting 30-day mortality after EL.

**Graphical Abstract:**

**Figure:**
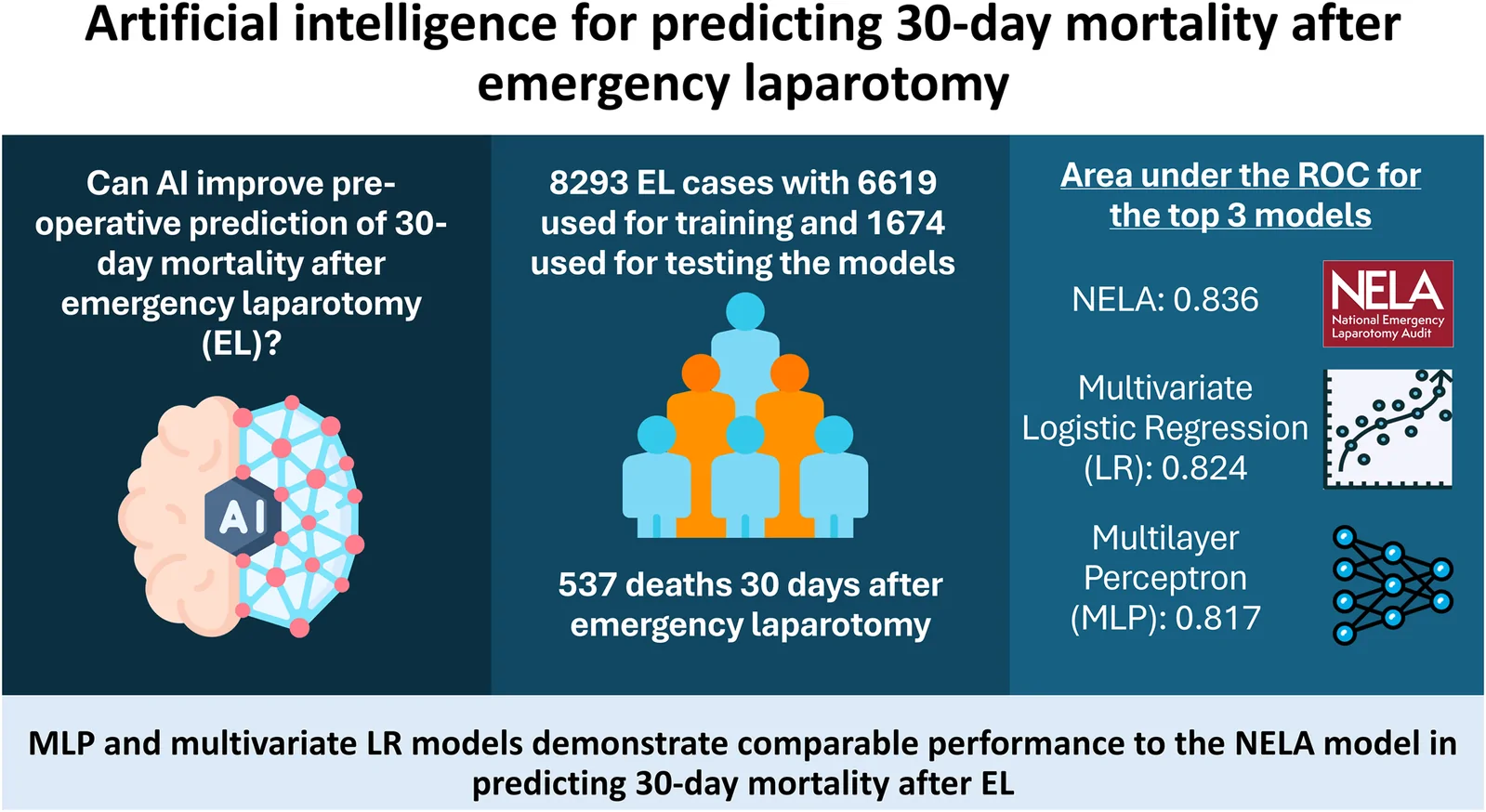

**Supplementary Information:**

The online version contains supplementary material available at https://doi.org/10.1007/s00068-026-03319-w.

## Introduction

Emergency laparotomies (ELs) are frequently lifesaving, however, can be associated with high mortality rates. In Australia and New Zealand (ANZ), approximately 20,000 ELs are performed annually, and emergency general surgical admissions are considerably more numerous [1–3]. The 30-day mortality rate following EL is estimated to be 8.4% [4]. Consequently, the Australian and New Zealand Emergency Laparotomy Audit – Quality Improvement (ANZELA-QI) database has been established to audit outcomes following EL and provide standardisation of the quality of care for patients, with the aim of reduced mortality rates [3, 5].

Standardising peri-operative EL care through quality improvement care bundles has been shown to reduce postoperative mortality [6]. In contemporary practice, this necessitates calculating and considering the risks associated with EL pre-operatively as this can lead to step-wise postoperative care escalation, identification of high-risk cases requiring greater consultant involvement, enhanced collaboration between surgical disciplines, and optimal shared decision-making with the patient [7–9]. Predictors of mortality in patients undergoing EL include advanced age, comorbidities, frailty, sarcopenia, American Society of Anesthesiologists (ASA) grade, and peritoneal contamination [10–12]. These predictors have led to the development of several risk prediction models to estimate postoperative mortality after EL [13]. Despite modern guidelines endorsing the use of risk prediction models, the optimal model remains unclear [14].

Despite the success of logistic regression (LR) models in predicting mortality, they are limited by assumptions of linear relationships between predictors and outcomes [15]. Artificial intelligence (AI) has gained popularity for its ability to leverage existing high-dimensional data and uncover complex non-linear relationships, providing a promising alternative to LR approaches [16]. Recent evidence shows that AI can achieve remarkably high performance in predicting mortality following emergency general surgical procedures and EL [17–19]. However, most AI models have been developed and validated in US populations and remain unvalidated in the Australasian setting. The National Emergency Laparotomy Audit (NELA) model developed in the UK has been extensively validated in Australian cohorts and remains the preferred risk assessment tool for postoperative mortality after EL [20, 21]. Nonetheless, the development of an ANZ model is warranted, given the distinct healthcare system of ANZ and the unique challenges posed by large geographical distances. In light of the paucity of local studies developing AI models to predict mortality following EL, this prognostic study aimed to use ANZELA-QI data to establish the accuracy of AI models in predicting death 30 days after EL in the Australasian population.

## Methods

### Ethics and reporting

This prognostic study was approved by the ANZELA-QI Steering Committee and adhered to the TRIPOD + AI statement [22]. ANZELA-QI has national ethics approval with waiver of patient consent by the South Metropolitan Health Service Human Research Ethics Committee (reference number: RGS0000000848). Each participating hospital then obtained local governance approval before data collection. ANZELA-QI is recognised as a Clinical Quality Registry with the Australian Commission of Safety and Quality in Health Care. The study was conducted in accordance with the principles of the 1964 Declaration of Helsinki and its later amendments.

### Data source

A complete extract of prospectively collected data from ANZELA-QI was obtained from the Royal Australasian College of Surgeons (RACS) Morbidity Audits Department of all patients who underwent an EL in 35 Australian hospitals between 1 July 2018 and 24 July 2023. The inclusion and exclusion criteria used by ANZELA-QI were similar to those used by the NELA [23, 24].

### Predictors

Predictors consisted of routinely collected pre-operative variables available in the ANZELA-QI dataset include age, sex, ethnicity, nature of admission, readmission within 30 days for a previous EL, emergency department admission, residence prior to hospital admission, transfer from another hospital, pre-operative diagnostic abdominal computed tomography (CT) scan performed, suspicion of sepsis at the time of initial admission, suspicion of sepsis at the time of decision for surgery, serum lactate level, ASA grade, surgical urgency, whether it was the first procedure within the admission, indication for surgery, most senior surgeon in theatre, and seniority of the anesthetist in theatre. Time from first arrival to surgical review, knife to skin, and administration of antibiotics were calculated for each patient and then stratified into <24 hours and ≥24 hours. A few categorical variables were recategorised to avoid modelling categories containing few patients or events. The recategorised variables included ethnicity, residence prior to hospital admission, ASA score, indications for surgery, most senior surgeon in theatre and most senior anesthetist in theatre (Supplementary Table 1).

### Outcome

The predicted outcome was 30-day mortality after EL. The elapsed time from the day of EL to the day of death was calculated for each patient who died. Patients that were discharged alive before 30 days after EL were assumed to have survived for at least 30 days. Patients who died within 30 days of EL were assigned to the non-survivors group, while those who survived beyond this threshold were assigned to the survivors group.

### Missing data

Missing data were replaced using multiple imputations by chained equations (MICE) [25]. This process involved creating five plausible imputations for each missing value and aggregating them by taking the mean for continuous variables and the mode for categorical values. In the primary analysis, MICE was performed before random allocation of ELs into training and testing datasets.

### Model development

The ANZELA-QI database was randomly split into two datasets in line with Pareto’s ratio (80% for model training and 20% for testing) to achieve better training and minimise model over- and under-fitting. We developed four artificial neural network-based AI models using multilayer perceptron (MLP) and radial basis function (RBF) architectures. Both networks are supervised, as model predictions can be compared with known values of the target variable, and employ a feedforward architecture, in which connections flow from the input layer to the output layer without feedback loops. Generally, MLP networks can handle more complex relationships, whereas RBF networks, which are characterised by a single hidden layer, are generally faster [26]. MLP and RBF models were run 30 times each to determine the optimal model. Simplified MLP and RBF models were built using statistically significant variables derived from univariate analysis, with 7 neurons in the MLP hidden layer and 10 neurons in the RBF layer. Extended MLP and RBF models not restricted to significant variables found in the univariate analysis were developed to estimate the extent to which the artificial neural networks could predict outcomes. The extended MLP and RBF models included all 25 pre-operative variables, 8 neurons in the MLP hidden layer and 10 neurons in the RBF layer. In addition to the AI models, a multivariate LR model was built using statistically significant variables derived from univariate analysis. The NELA model is a well-recognized risk prediction calculator for 30-day mortality after EL, and is the tool used to predict the risk of mortality for each patient in the ANZELA-QI dataset. For our analysis, these risk percentages were converted to probabilities. The predicted probabilities from NELA were then compared with the output values generated by the AI and multivariate LR models.

A complete-case sensitivity analysis was performed to assess whether performing MICE before dataset splitting influenced model performance. Patients with missing predictor data were excluded before random allocation into training and testing cohorts, and models were developed using the same input parameters as the primary analysis.

### Statistical analysis

Parametricity was tested using the Kolmogorov-Smirnov test, and results are presented as mean (standard deviation) for parametric and median (interquartile range [IQR]) for nonparametric data. Categorical variables are displayed as frequency (percentage). Discrimination of the different models was assessed using the area under the receiver operating characteristic curve (AUROC). Youden’s index was established for 30-day mortality to identify the optimal dichotomous cutoff to determine sensitivity, specificity, positive predictive value, negative predictive value, positive likelihood ratio, negative likelihood ratio and the overall percentage of ELs correctly classified. Differences in performance between models were assessed with the DeLong test for the difference in AUROC [27]. Univariate analysis was conducted using Pearson’s χ^2^ test for categorical variables and Student T-test or Mann-Whitney U-Test for continuous variables, depending on the type of distribution. To assess how well the multivariate LR model fits the data, a goodness of fit was evaluated using Pearson’s χ^2^ test. An independent variable importance analysis was performed to evaluate the extent to which MLP and RBF models relied on specific variables to make accurate predictions. Wald statistics was examined to identify the most important predictor for the multivariate LR model. AUROC values from the primary MICE-imputed analysis were compared descriptively with those from the complete-case analysis. All metrics are reported with 95% confidence intervals (CIs), and 2-tailed tests were considered statistically significant at *p* < 0.05. Data were analysed using IBM SPSS Statistics for Windows Version 27.0 (IBM Corp, Armonk, NY, USA) and MedCalc Statistical Software version 22 (MedCalc Software bv, Ostend, Belgium; https://www.medcalc.org; 2020). Calibration was assessed in the testing dataset using R (Version 4.6.1; R Core Team, 2026) and RStudio IDE (Version 2026.07.1; Posit Team, 2026). Calibration plots were generated by comparing predicted and observed 30-day mortality across deciles of predicted risk. Calibration intercepts, calibration slopes and Brier scores were calculated for each model. For validation purposes, SPSS calculators and instructions on how to use the five models developed in this study are provided in the Supplementary Methods in XML format.

## Results

A total of 8,293 ELs were identified during the study period, with 537 (6.5%) deaths within 30 days. EL procedures are detailed in the Supplementary Material (Supplementary Table 2). The training and testing datasets consisted of 6619 (80%) and 1674 (20%) ELs, respectively (Supplementary Figure 1). Baseline patient characteristics before multpiple imputations are available in Supplementary Table 3, and after imputations are presented in Table 1 and Supplementary Table 4. Median age was 66 years (IQR 52–77), and 50.7% were females. Univariate analysis showed a significant difference in 30-day postoperative mortality for 17 variables: age, sex, residence prior to hospital admission, transfer from another hospital, time from first arrival to first seen by any member of the surgical team, pre-operative diagnostic abdominal CT scan performed, suspicion of sepsis at the time of initial admission, suspicion of sepsis at the time of decision for surgery, lactate, ASA, urgency, first procedure within this admission, four surgical indications (bleeding, obstruction, ischaemia, other) and most senior surgeon in theatre (all *p* < 0.05). Multivariate LR analysis identified age, pre-operative diagnostic abdominal CT scan performed, suspicion of sepsis at the time of decision for surgery, lactate, ASA, urgency, obstruction, ischemia and other indications as independent predictors of 30-day postoperative mortality (all *p* < 0.05) (Supplementary Table 5).

**Table 1:** Baseline patient characteristics following replacement of missing data with multiple imputation

Variable	Total (n = 8293)	Survivors (n = 7756)	Non-survivors (n = 537)	p value
**Age (years), median (IQR)**	66 (52–77)	65 (52–76)	74 (64–82)	**<0.001**
**Sex**				**0.020**
Male	4091 (49.3)	3800 (49.0)	291 (54.2)	
Female	4202 (50.7)	3956 (51.0)	246 (45.8)	
**Ethnicity**				0.294
Indigenous	1527 (18.4)	1419 (18.3)	108 (20.1)	
Any other ethnicity	6766 (81.6)	6337 (81.7)	429 (79.9)	
**Admission type**				0.111
Elective	447 (5.4)	410 (5.3)	37 (6.9)	
Emergency	7846 (94.6)	7346 (94.7)	500 (93.1)	
**Readmission within 30 days for a previous EL**				0.996
Yes	201 (2.4)	188 (2.4)	13 (2.4)	
No	8092 (97.6)	7568 (97.6)	524 (97.6)	
**Admitted via ED**				0.062
Yes	7369 (88.9)	6905 (89.0)	464 (86.4)	
No	924 (11.1)	851 (11.0)	73 (13.6)	
**Residence prior to hospital admission**				**<0.001**
Own home	7878 (95.0)	7390 (95.3)	488 (90.9)	
Other	415 (5.0)	366 (4.7)	49 (9.1)	
**Transfer from another hospital**				**0.048**
Yes	1905 (23.0)	1763 (22.7)	142 (26.4)	
No	6388 (77.0)	5993 (77.3)	395 (73.6)	
**Time from first arrival to first seen by any member of surgical team**				**0.008**
<24 hours	6350 (76.6)	5964 (76.9)	386 (71.9)	
≥24 hours	1943 (23.4)	1792 (23.1)	151 (28.1)	
**Time from first arrival to knife to skin**				0.920
<24 hours	2933 (35.4)	2742 (35.4)	191 (35.6)	
≥24 hours	5360 (64.6)	5014 (64.6)	346 (64.4)	
**Time from first arrival to administration of antibiotics**				0.822
<24 hours	4656 (56.1)	4352 (56.1)	304 (56.6)	
≥24 hours	3637 (43.9)	3404 (43.9)	233 (43.4)	
**Preoperative diagnostic abdominal CT scan performed**				**<0.001**
Yes	7682 (92.6)	7206 (92.9)	476 (88.6)	
No	611 (7.4)	550 (7.1)	61 (11.4)	
**Suspicion of sepsis at the time of initial admission**				**<0.001**
Yes	2295 (27.7)	2102 (27.1)	193 (35.9)	
No	5998 (72.3)	5654 (72.9)	344 (64.1)	
**Suspicion of sepsis at the time of decision for surgery**				**<0.001**
Yes	2821 (34.0)	2547 (32.8)	274 (51.0)	
No	5472 (66.0)	5209 (67.2)	263 (49.0)	
**Lactate (mmol/L), median (IQR)**	1.9 (1.3–2.7)	1.9 (1.2–2.7)	2.7 (1.6–4.5)	**<0.001**
**ASA score**				**<0.001**
<3	2686 (32.4)	2650 (34.2)	36 (6.7)	
≥3	5607 (67.6)	5106 (65.8)	501 (93.3)	
**Urgency (hours), median (IQR)**	6 (4–12)	6 (4–12)	4 (2–6)	**<0.001**
**First procedure within this admission**				**<0.001**
Yes	7361 (88.8)	6925 (89.3)	436 (81.2)	
No	932 (11.2)	831 (10.7)	101 (18.8)	
**Indication for surgery**				
Bleeding	412 (5.0)	374 (4.8)	38 (7.1)	**0.020**
Obstruction	4534 (54.7)	4342 (56.0)	192 (35.8)	**<0.001**
Sepsis	3008 (36.3)	2794 (36.0)	214 (39.9)	0.074
Ischaemia	897 (10.8)	753 (9.7)	144 (26.8)	**<0.001**
Other	107 (1.3)	87 (1.1)	20 (3.7)	**<0.001**
**Most senior surgeon in theatre**				**0.010**
Consultant surgeon	8197 (98.8)	7660 (98.8)	537 (100.0)	
Trainee surgeon	96 (1.2)	96 (1.2)	0 (0.0)	
**Most senior anesthetist in theatre**				0.064
Consultant anesthetist	7802 (94.1)	7287 (94.0)	515 (95.9)	
Trainee anesthetist	491 (5.9)	469 (6.0)	22 (4.1)	

Values are n (%) unless otherwise indicated

ASU, acute surgical unit; CT, computed tomography; NELA, national emergency laparotomy audit; ASA, American Society of Anaesthesiologists; EL, emergency laparotomy

### Model performance

**Fig. 1:**
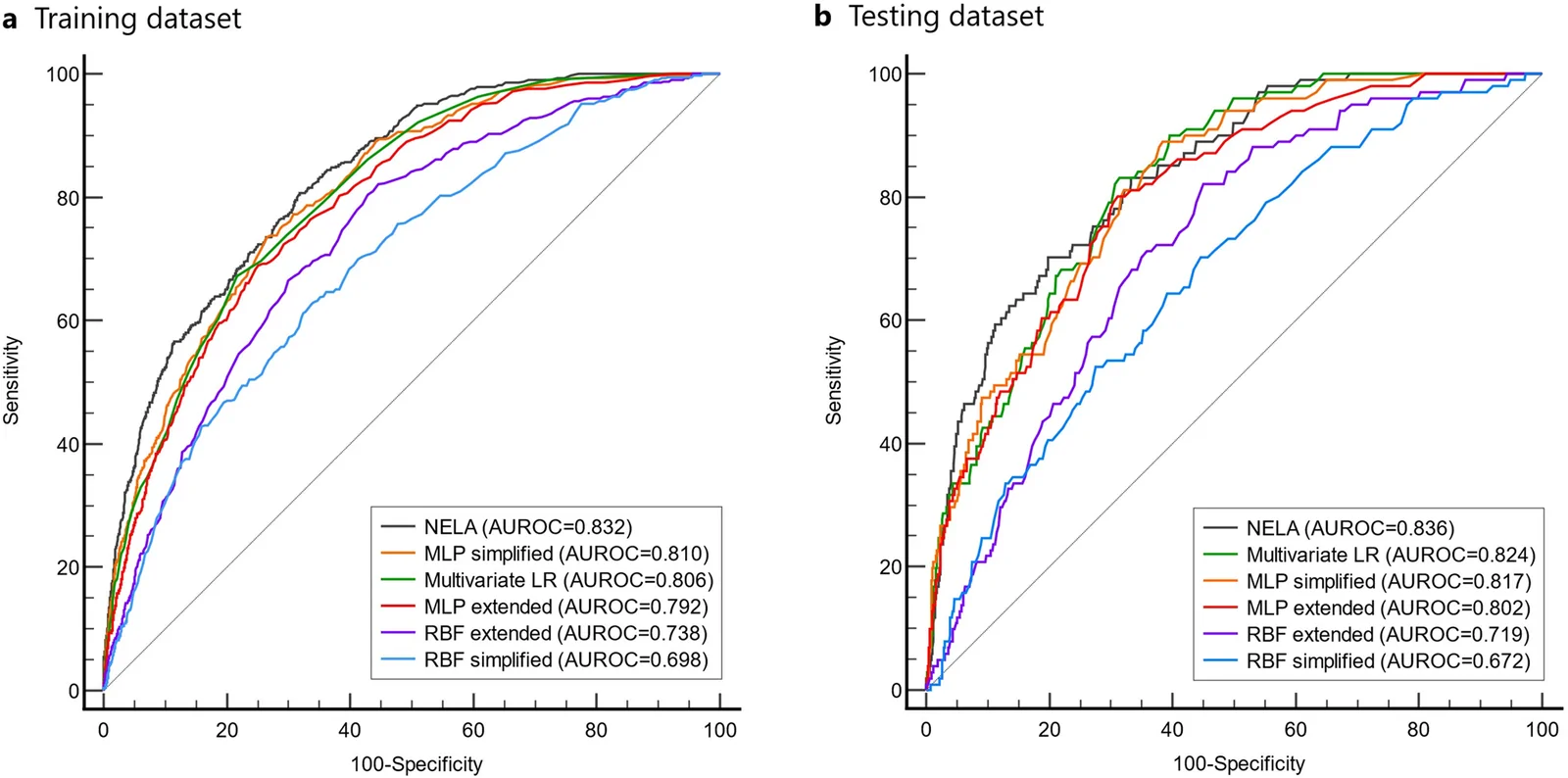
Receiver operating characteristic curves for models in **a** training dataset and **b** testing dataset

Discrimination was assessed in the training and testing datasets (Fig. 1, Table 2). AUROC values for predicting mortality for NELA, MLP simplified, multivariate LR, MLP extended, RBF extended, and RBF simplified models in the training dataset were 0.832 (95% CI, 0.815 to 0.850), 0.810 (95% CI, 0.791 to 0.829), 0.806 (95% CI, 0.788 to 0.825), 0.792 (95% CI, 0.772 to 0.812), 0.738 (95% CI, 0.715 to 0.761) and 0.698 (95% CI, 0.673 to 0.723), respectively. In the testing dataset, the AUROC for the NELA, multivariate LR, MLP simplified, MLP extended, RBF extended, and RBF simplified models were 0.836 (95% CI, 0.799 to 0.872), 0.824 (95% CI, 0.790 to 0.858), 0.817 (95% CI, 0.781 to 0.854), 0.802 (95% CI, 0.762 to 0.843), 0.719 (95% CI, 0.674 to 0.764) and 0.672 (95% CI, 0.620 to 0.724), respectively.

**Table 2:** Model discrimination

Models	AUROC	Sensitivity, %	Specificity, %	PLR	NLR	PPV,%	NPV, %	Correctly classified
**Training (n = 6619)**								
NELA	0.832 (0.815, 0.850)	80.5 (76.5, 84.1)	68.1 (66.9, 69.3)	2.5 (2.4, 2.7)	0.3 (0.2, 0.4)	15.1 (14.4, 15.9)	98.0 (97.6, 98.4)	68.9 (67.8, 70.0)
MLP simplified	0.810 (0.791, 0.829)	73.6 (69.2, 77.7)	73.4 (72.3, 74.5)	2.8 (2.6, 3.0)	0.4 (0.3, 0.4)	16.3 (15.4, 17.3)	97.5 (97.1, 97.9)	73.4 (72.3, 74.5)
Multivariate LR	0.806 (0.788, 0.825)	67.2 (62.6, 71.6)	78.4 (77.3, 79.4)	3.1 (2.9, 3.4)	0.4 (0.4, 0.5)	18.0 (16.8, 19.2)	97.1 (96.7, 97.5)	77.7 (76.6, 78.7)
MLP extended	0.792 (0.772, 0.812)	69.0 (64.5, 73.3)	74.8 (73.7, 75.9)	2.7 (2.5, 3.0)	0.4 (0.4, 0.5)	16.2 (15.2, 17.2)	97.2 (96.7, 97.5)	74.4 (73.4, 75.5)
RBF extended	0.738 (0.715, 0.761)	80.5 (76.5, 84.1)	57.1 (55.9, 58.4)	1.9 (1.8, 2.0)	0.3 (0.3, 0.4)	11.7 (11.1, 12.3)	97.6 (97.2, 98.1)	58.6 (57.4, 59.8)
RBF simplified	0.698 (0.673, 0.723)	62.8 (58.1, 67.4)	66.2 (65.0, 67.3)	1.9 (1.7, 2.0)	0.6 (0.5, 0.6)	11.6 (10.8, 12.4)	96.2 (95.7, 96.6)	66.0 (64.8, 67.1)
**Testing (n = 1674)**								
NELA	0.836 (0.799, 0.872)	70.3 (60.4, 79.0)	80.2 (78.2, 82.2)	3.6 (3.0, 4.2)	0.4 (0.3, 0.5)	18.6 (16.3, 21.1)	97.7 (96.9, 98.3)	79.6 (77.6, 81.5)
Multivariate LR	0.824 (0.790, 0.858)	83.2 (74.4, 89.9)	68.5 (66.2, 70.8)	2.6 (2.4, 3.0)	0.3 (0.2, 0.4)	14.5 (13.1, 16.0)	98.4 (97.6, 99.0)	69.4 (67.1, 71.6)
MLP simplified	0.817 (0.781, 0.854)	89.1 (81.3, 94.4)	61.6 (59.1, 64.0)	2.3 (2.1, 2.6)	0.2 (0.1, 0.3)	13.0 (12.0, 14.0)	98.9 (98.1, 99.4)	63.2 (60.9, 65.6)
MLP extended	0.802 (0.762, 0.843)	80.2 (71.1, 87.5)	68.8 (66.4, 71.1)	2.6 (2.3, 2.9)	0.3 (0.2, 0.4)	14.2 (12.7, 15.7)	98.2 (97.3, 98.8)	69.5 (67.2, 71.7)
RBF extended	0.719 (0.674, 0.764)	82.2 (73.3, 89.1)	54.9 (52.4, 57.4)	1.8 (1.6, 2.0)	0.3 (0.2, 0.5)	10.5 (9.5, 11.5)	98.0 (96.9, 98.7)	56.6 (54.2, 59.0)
RBF simplified	0.672 (0.620, 0.724)	70.3 (60.4, 79.0)	55.3 (52.8, 57.8)	1.6 (1.4, 1.8)	0.5 (0.4, 0.7)	9.2 (8.1, 10.4)	96.7 (95.5, 97.5)	56.2 (53.8, 58.6)

Values in parentheses are 95% confidence intervals

AUROC, area under the receiver operating characteristics curve; PPV, positive predictive value; NPV, negative predictive value; PLR, positive likelihood ratio; NLR, negative likelihood ratio; RBF, radial basis function; MLP, multilayer perceptron; LR, logistic regression; NELA, national emergency laparotomy audit risk calculator

Using the maximum Youden’s index, cut-off was highest in NELA (79.6%), followed by MLP extended (69.5%), multivariate LR (69.4%), MLP simplified (63.2%), RBF extended (56.6%), and RBF simplified models (56.2%), for ELs in the testing dataset being correctly classified for 30-day postoperative mortality. The model with the highest sensitivity was MLP simplified (89.1%), followed by multivariate LR (83.2%), RBF extended (82.2%), MLP extended (80.2%), NELA (70.3%) and RBF simplified (70.3%). Specificity was highest in NELA (80.2%), followed by MLP extended (68.8%), multivariate LR (68.5%), MLP simplified (61.6%), RBF simplified (55.3%), and RBF extended (54.9%).

### Comparison of model performance

Pairwise comparison of models on the testing dataset is shown in Table 3. The AUROC for NELA, multivariate LR, MLP simplified, and MLP extended models were significantly greater than those for RBF simplified and RBF extended models. The RBF extended model had a significantly higher AUROC compared to the RBF simplified model. There was no significant difference in AUROC values among the NELA, multivariate LR, MLP simplified and MLP extended models.

**Table 3:** Pairwise comparison of ROC curves on the testing dataset

Comparison	Difference between areas	SE	p value
NELA vs Multivariate LR	0.011 (−0.030, 0.052)	0.021	0.588
NELA vs MLP simplified	0.018 (−0.024, 0.060)	0.022	0.399
NELA vs MLP extended	0.033 (−0.013, 0.079)	0.024	0.159
NELA vs RBF extended	0.117 (0.066, 0.167)	0.026	**<0.001**
NELA vs RBF simplified	0.163 (0.104, 0.223)	0.030	**<0.001**
Multivariate LR vs MLP simplified	0.0068 (−0.006, 0.020)	0.006	0.294
Multivariate LR vs MLP extended	0.022 (−0.0027, 0.046)	0.013	0.082
Multivariate LR vs RBF extended	0.105 (0.066, 0.145)	0.020	**<0.001**
Multivariate LR vs RBF simplified	0.152 (0.105, 0.200)	0.024	**<0.001**
MLP simplified vs MLP extended	0.0150 (−0.0136, 0.0436)	0.0146	0.304
MLP simplified vs RBF extended	0.099 (0.058, 0.139)	0.021	**<0.001**
MLP simplified vs RBF simplified	0.145 (0.097, 0.194)	0.025	**<0.001**
MLP extended vs RBF extended	0.084 (0.034, 0.133)	0.025	**0.0009**
MLP extended vs RBF simplified	0.130 (0.078, 0.182)	0.027	**<0.001**
RBF extended vs RBF simplified	0.047 (0.008, 0.086)	0.020	**0.018**

Values in parentheses are 95% confidence intervals

RBF, radial basis function; MLP, multilayer perceptron; LR, logistic regression; NELA, national emergency laparotomy audit risk calculator; SE, standard error

### Variable importance

**Fig. 2:**
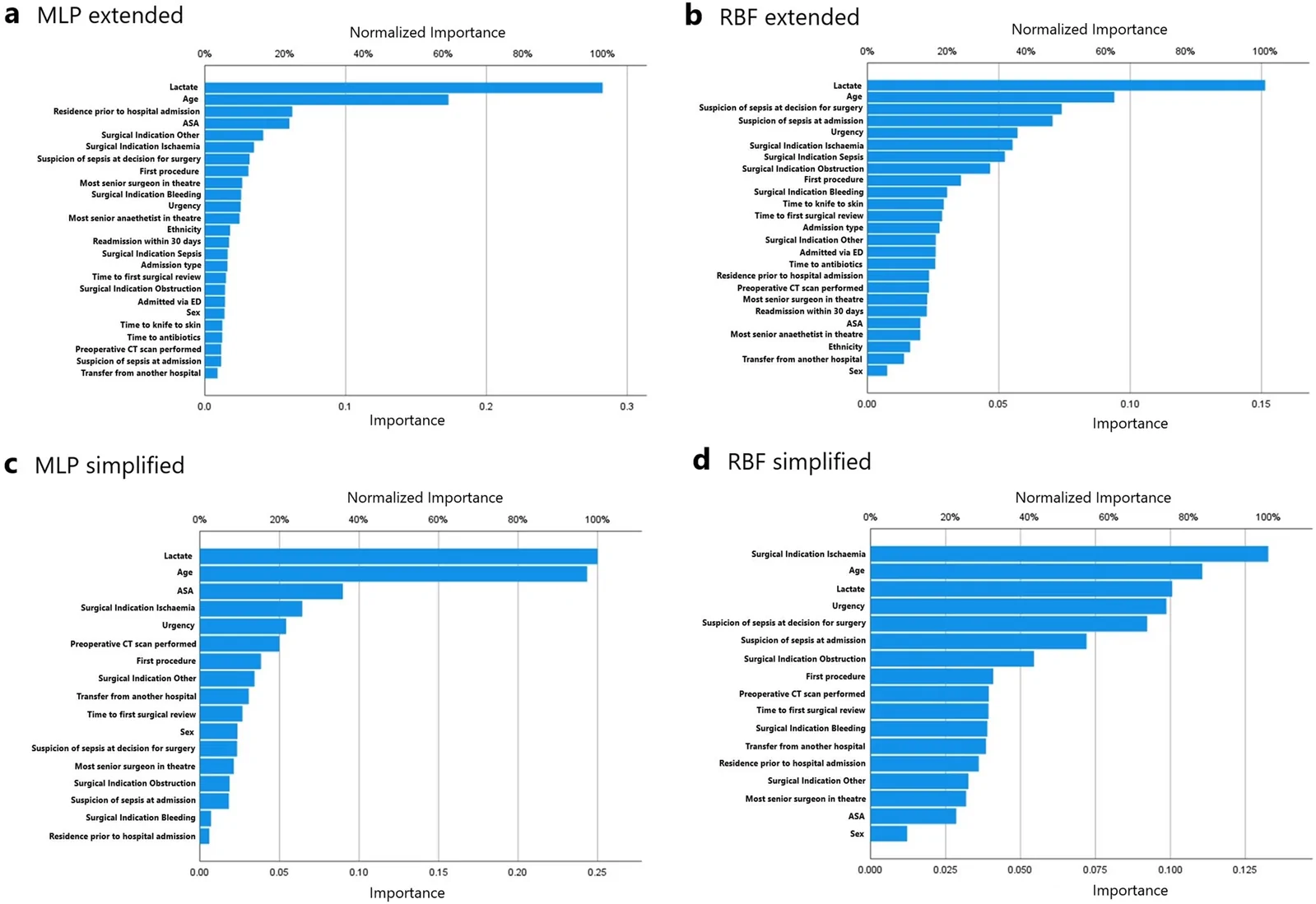
Weighted importance of included variables for each model. Both variable importance and relative weight of the predictive models shifted as indicated on the x-axis of each graph. **a** MLP extended model; **b** RBF extended model; **c** MLP simplified model and **d** RBF simplified model

Multivariate LR analysis found age to be the most critical predictor, with a Wald of 92.84 (Supplementary Table 6). The most influential predictor variable in the MLP extended, RBF extended, and MLP simplified models was lactate, followed by age (Supplementary Table 6). The most influential predictor variable in the RBF simplified model was ischemia, followed by lactate. Overall, there was considerable variability in importance across models. The normalised variable importance graphs are presented in Fig. 2.

### Sensitivity analysis

A complete-case sensitivity analysis was performed among 2944 ELs without missing data. The training and testing datasets consisted of 2356 (80%) and 588 (20%) ELs, respectively. Deaths within 30 days occurred in 178 (7.6%) ELs in the training dataset and 45 (7.7%) ELs in the testing dataset. Overall, model discrimination was similar to the primary MICE-imputed analysis (Supplementary Table 7). The NELA model demonstrated comparable performance between analyses in both the training dataset (AUROC 0.841 vs 0.832) and testing dataset (AUROC 0.828 vs 0.836). Similar trends were observed for the multivariate LR model, while the extended and simplified MLP and RBF models demonstrated greater variability, likely reflecting a reduced sample size and fewer outcome events in the complete-case dataset.

### Calibration

**Fig. 3:**
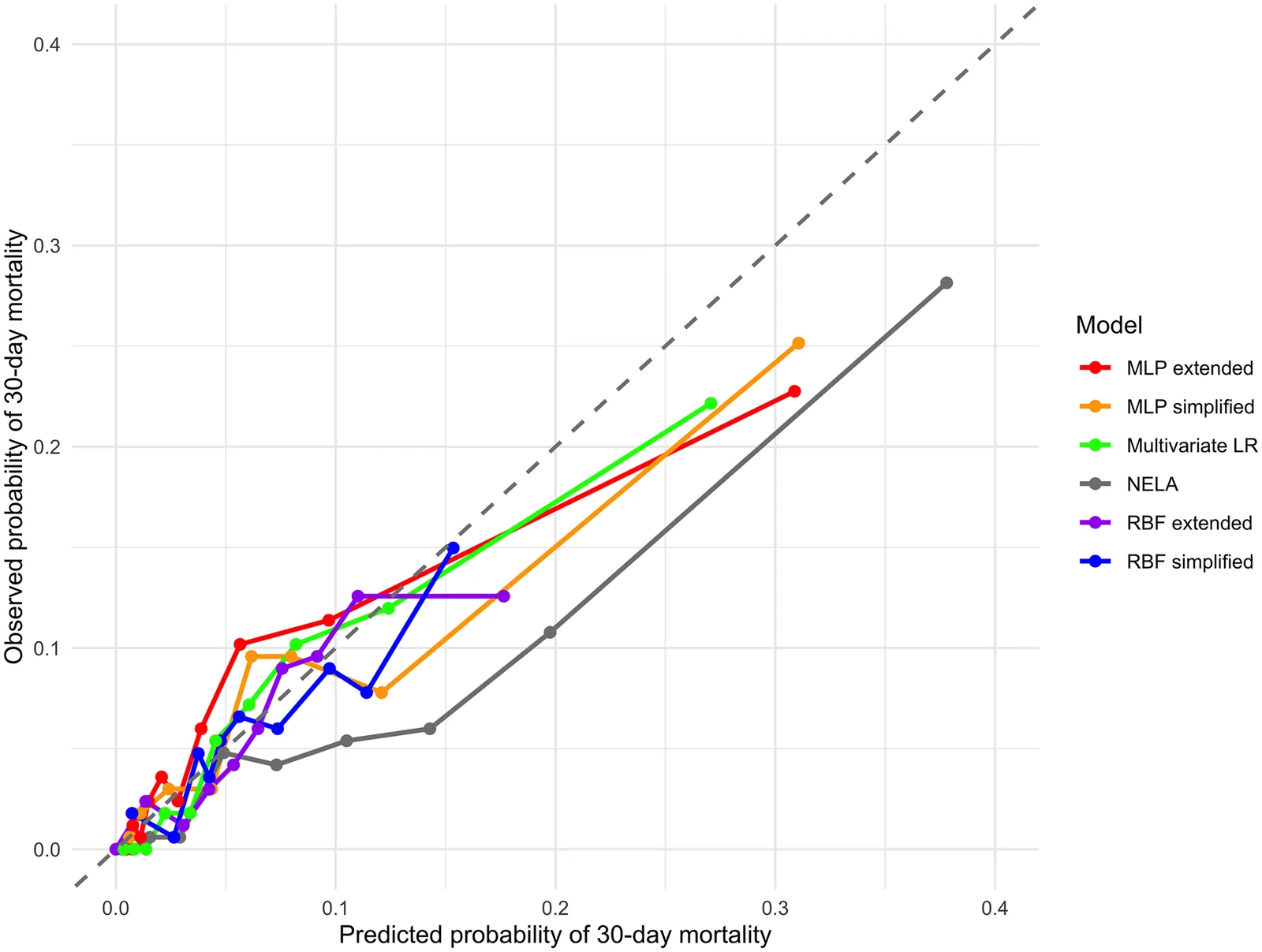
Calibration plots for prediction of 30-day mortality in the training dataset

Calibration was assessed in the testing dataset (Supplementary Table 8). Calibration performance varied between models (Fig. 3). The multivariate LR model demonstrated a calibration intercept of −0.104 (95% CI, −0.567 to 0.359) and calibration slope of 1.005 (95% CI, 0.811 to 1.200), indicating good calibration. NELA demonstrated a calibration slope of 1.014 (95% CI, 0.819 to 1.209), although its calibration intercept was −0.616 (95% CI, −0.979 to −0.254). The multivariate LR and simplified MLP models had the lowest Brier scores (0.051), while the RBF models had lower calibration slopes, particularly the simplified RBF model, which had a calibration slope of 0.559 (95% CI, 0.264 to 0.854).

## Discussion

This is the first study to employ artificial neural network-based AI models to predict 30-day mortality after EL in Australian patients. The performance of four AI models, a multivariate LR model and the existing NELA model were examined to predict individual probabilities of 30-day mortality after EL. Pairwise comparisons revealed no significant difference in the predictive performance of the tested models. Furthermore, a complete-case sensitivity analysis demonstrated similar discrimination compared with the primary MICE-imputed analysis, suggesting that the reported model performance was not substantially influenced by the imputation strategy. Notably, despite both the multivariate LR and MLP models being developed using less source data than the original NELA dataset, their AUROC values were comparable.

There are several prediction models available for estimating 30-day mortality after EL [13]. These models are crucial in facilitating shared, evidence-informed decision-making regarding the appropriateness of proceeding with EL and managing patient and family expectations. Comparative studies that have externally validated these risk prediction models in ANZ have mostly favoured the NELA model [20, 21, 28]. In contrast, our study found no statistically significant difference in the discriminatory power of the NELA compared to the multivariate LR and MLP models developed from ANZELA-QI (AUROC = 0.836 vs 0.824 vs 0.817). The slightly lower AUROC value of the multivariate LR and MLP models might be attributed to their development in a smaller patient cohort. Notably, the NELA model was developed on 38,830 patients undergoing EL, well above the 8,293 ELs used in developing our models. Furthermore, the NELA risk adjustment model for 30-day mortality after EL was developed to facilitate case-mix-adjusted comparisons of mortality rates between hospitals and was not intended to predict individual patient risk within a clinical setting [29]. Additionally, some variables in the NELA model are only available during or after surgery, limiting its pre-operative utility.

To address this issue, the NELA project team recently developed a model that utilises several pre-operative variables to predict individual probabilities of mortality 30 days after EL [30]. The pre-operative NELA model includes 13 variables and demonstrated high discrimination during testing (AUROC = 0.86). This model’s excellent performance can be attributed to the inclusion of routinely recorded hemodynamic parameters and laboratory tests, which strongly reflect the severity of the underlying abdominal pathology and the patient’s physical condition. The recently published HAS model incorporated the Hajibandeh Index, ASA status and sarcopenia to predict 30-day mortality following EL. The HAS model presented superior discrimination (AUROC = 0.96) compared to the pre-operative NELA model and our models [31]. Future models could benefit from including hemodynamic and laboratory parameters to enhance accuracy and applicability.

Recent advancements in AI have led to the development of three predictive models for postoperative mortality after EL: two based on the American College of Surgeons National Surgical Quality Improvement Program (ACS NSQIP) data, and one using the ANZELA-QI dataset [17, 18, 32]. The Predictive Optimal Trees in Emergency Surgery Risk (POTTER) model was initially designed to predict mortality in emergency surgery patients but has since been prospectively validated in patients undergoing emergency general surgery and EL, achieving an AUROC of 0.90 [33]. Gao et al. developed an artificial neural network model to predict mortality of emergency general surgery patients exceptionally well, with an impressive AUROC of 0.956, however, external validation is required [17]. Although these models demonstrated superior performance to our MLP simplified model (AUROC = 0.817), the pragmatic utilisation of only 17 pre-operative variables makes our model more user-friendly and clinically relevant. More recently, Jones et al. developed pre-operative and intra-operative mortality prediction models for EL using LR, random forest, and XGBoost on the ANZELA-QI dataset [32]. In comparison, both our multivariate LR and simplified MLP models outperformed those reported by Jones et al. Further research is warranted to strike an optimal balance between model performance, simplicity, and the feasibility of implementing AI-based decision support tools in clinical practice.

Our findings revealed serum lactate and age as the most important variables across the five models. Lactate levels have been established as a predictor of mortality in EL patients [34]. Our study confirmed that median lactate levels were significantly raised in non-survivors compared with survivors after EL (2.7 vs 1.9 mmol/L), making it a valuable predictor of mortality 30 days after EL. Moreover, age has been linked as an independent risk factor for mortality in EL patients, likely due to the increasing prevalence of comorbidities, sarcopenia, and frailty among older patients [35, 36]. Since the HAS model incorporates sarcopenia as one of its predictor variables, it is evident that including sarcopenia or frailty could enhance the performance of our models [31]. However, the diagnostic criteria for sarcopenia requires a comprehensive evaluation of muscle quality, strength and function, which is time-consuming and may not be practical in the emergency setting [37].

As the volume of EL continues to grow worldwide, identifying high-risk patients becomes increasingly important for guiding clinical decision-making in the pre-operative period [14, 38]. Currently, when patients and their families inquire about the risk of an EL, surgeons often rely on anecdotal evidence instead of evidence-based risk prediction models, likely reflecting the complexity of decision making and risk prediction in emergency settings [39]. In this era of personalised medicine, using a multivariate LR or simplified AI model, such as the ones we developed, offers an opportunity to provide precise individualised mortality predictions after EL. This could serve as an invaluable aid to decision making for surgeons, patients, and their families, in what is often a challenging and time sensitive situation. Furthermore, considering the poor uptake for risk assessment in EL patients across some hospitals, the models developed in this study have the potential to be integrated into Electronic Medical Records systems, improving clinician uptake and offering an individualised prediction of 30-day mortality that is readily available [40]. It is important to note that some of the variables in our models relied on clinical judgement, such as suspicion of sepsis at the time of initial admission, suspicion of sepsis at the time of decision for surgery, indication for surgery, and ASA status. The categorisation of these variables might vary between hospitals. Nonetheless, most variables in the model are commonly collected and have agreed-upon definitions, maintaining generalisability across different hospitals.

This study has several limitations. Firstly, participation in ANZELA-QI accounts for only 10–20% of EL performed. Secondly, ANZELA-QI was only established in 2018, leading to its small sample size compared to other registries such as NELA and NSQIP. Thirdly, missing data were handled using multiple imputation prior to dataset splitting, which may have introduced bias and potential information leakage. However, the sensitivity analysis restricted to complete cases demonstrated comparable model discrimination, suggesting that any potential impact of the imputation strategy on model performance was likely limited. Lastly, an external validation cohort is lacking, necessitating cautious interpretation of the results outside of the Australasian population.

## Conclusion

Pre-operative multivariate LR and MLP models demonstrated comparable performance to the NELA model in predicting 30-day mortality after EL. Additionally, AI-based MLP models offered no significant discriminatory advantage over a multivariate LR model, suggesting that the LR model may be the preferred, more transparent tool for clinical integration. Further external validation studies are required to substantiate their clinical utility.

## Electronic supplementary material

Below is the link to the electronic supplementary material.


Supplementary material 1


## Data Availability

The ANZELA-QI data will not be available to third parties.
