# Supplementary material for: Artificial intelligence for predicting 30-day mortality after emergency laparotomy
Source: Eur J Trauma Emerg Surg. 2026 Aug 31;52(1):273. doi: 10.1007/s00068-026-03319-w (PMC13529842; doi:10.1007/s00068-026-03319-w)
Supplement: Supplementary file 1 [file 68_2026_3319_MOESM1_ESM.docx]

**Supplementary Material**

**Table of contents**

**Supplementary Methods.** Instructions for SPSS calculators

**Supplementary Table and Figures**

**Figure S1.** Study flowchart

**Figure S2.** Overall model quality in testing cohort. A good model has a value above 0.5, a value less than 0.5 indicates the model is no better than random prediction.

**Table S1.** Reclassified variables

**Table S2.** Baseline patient characteristics with missing data before multiple imputations

**Table S3.** Baseline patient characteristics and primary outcome

**Table S4.** Multivariate logistic regression

**Table S5.** Variable importance

**Table S6.** Procedures

**Table S7** Sensitivity analysis assessing model discrimination using complete-case analysis without multiple imputation

**Table S8** Calibration performance in the testing dataset

**Supplementary Methods. Instructions for SPSS calculators**

In order for the multivariate LR SPSS calculator to work the variables in your dataset should be as follows: Sex (1=male/ 2=female), Time_to_first_surgical_review (1=less than 24 hours/ 2= more or equal to 24 hours), Age (years), Residence_prior_to_hospital_admission (1=Own home/ 2=Other residence), Transfer_from_another_hospital (1=yes/ 2=no), Preop_CT_scan_performed (1=yes/ 2=no), Lactate (mmol/L), Suspicion_of_sepsis_at_admission (1=yes/ 2=no), Suspicion_of_sepsis_at_decision_for_surgery (1=yes/ 2=no), ASA (1= less than 3/ 2= more or equal to 3), Urgency (hours), First_procedure (1=yes/ 2=no), Surgical_Indication_Bleeding (1=yes/ 2=no), Surgical_Indication_Other (1=yes/ 2=no), Surgical_Indication_Obstruction (1=yes/ 2=no), Surgical_Indication_Ischaemia (1=yes/ 2=no).

In order for the simplified MLP and RBF SPSS calculators to work the variables in your dataset should be as follows: Sex (1=male/ 2=female), Time_to_first_surgical_review (1=less than 24 hours/ 2= more or equal to 24 hours), Age (years), Residence_prior_to_hospital_admission (1=Own home/ 2=Other residence), Transfer_from_another_hospital (1=yes/ 2=no), Preop_CT_scan_performed (1=yes/ 2=no), Lactate (mmol/L), Suspicion_of_sepsis_at_admission (1=yes/ 2=no), Suspicion_of_sepsis_at_decision_for_surgery (1=yes/ 2=no), ASA (1= less than 3/ 2= more or equal to 3), Urgency (hours), First_procedure (1=yes/ 2=no), Surgical_Indication_Bleeding (1=yes/ 2=no), Surgical_Indication_Other (1=yes/ 2=no), Surgical_Indication_Obstruction (1=yes/ 2=no), Surgical_Indication_Ischaemia (1=yes/ 2=no), Most_senior_surgeon_in_theatre (1=consultant surgeon/ 2=trainee surgeon).

In order for the extended MLP and RBF SPSS calculators to work the variables in your dataset should be as follows: Sex (1=male/ 2=female), Ethnicity (1= indigenous/ 2= any other ethnicity), Time_to_first_surgical_review (1=less than 24 hours/ 2= more or equal to 24 hours), Time_to_knife_to_skin (1=less than 24 hours/ 2= more or equal to 24 hours), Time_to_antibiotics (1=less than 24 hours/ 2= more or equal to 24 hours), Age (years), Admission_type (1= elective/ 2=emergency), Readmission_within_30_days (1=yes/ 2=no), Admitted_via_ED (1=yes/ 2=no), Residence_prior_to_hospital_admission (1=Own home/ 2=Other residence), Transfer_from_another_hospital (1=yes/ 2=no), Preop_CT_scan_performed (1=yes/ 2=no), Lactate (mmol/L), Suspicion_of_sepsis_at_admission (1=yes/ 2=no), Suspicion_of_sepsis_at_decision_for_surgery (1=yes/ 2=no), ASA (1= less than 3/ 2= more or equal to 3), Urgency (hours), First_procedure (1=yes/ 2=no), Surgical_Indication_Bleeding (1=yes/ 2=no), Surgical_Indication_Other (1=yes/ 2=no), Surgical_Indication_Obstruction (1=yes/ 2=no), Surgical_Indication_Sepsis (1=yes/ 2=no), Surgical_Indication_Ischaemia (1=yes/ 2=no), Most_senior_surgeon_in_theatre (1=consultant surgeon/ 2=trainee surgeon), Most_senior_anaethetist_in_theatre (1=consultant anaesthetist / 2=trainee anaesthetist).

To use the desired model (multivariate LR, simplified MLP, simplified RBF, extended MLP and extended RBF), open SPSS and select your dataset that contains the various baseline characteristics of your patients. From the menus choose: **Utilities > Scoring Wizard.** Then, click **Browse** to navigate the location where you saved the desired model’s xml file and click **Select** in the Browse dialog. Select the desired model’ xml file and then click **Next**. In order to score your dataset, the dataset must contain variables that correspond to all predictors in the model (described above). By default, any variables in your dataset that have the same name as the variables in the model are automatically matched. Use the drop-down list to match the dataset variables to model variables. The data type for each variable must be the same in both the model and the dataset in order to match variables. Your dataset does not contain a variable named Mortality_30days, therefore the cell in the Dataset Fields column that corresponds to the model field Mortality_30days is initially blank. Check the **Probability of the Selected Category** and **Select 1** from the drop-down list. The list of possible values for Mortality_30days is defined as 0 for alive and 1 for dead. Uncheck all the other scoring functions. Click **Finish** to apply the desired model to your dataset. The new variable that contains the probability of a positive response is appended to the end of your dataset.

**MLP extended xml code**

**MLP simplified xml code**

**Multivariate LR xml code**

**RBF extended xml code**

**RBF simplified xml code**

**Supplementary Figure 1. Study flowchart**

**Supplementary Figure 2. Overall model quality in testing cohort. A good model has a value above 0.5, a value less than 0.5 indicates the model is no better than random prediction.**

**Supplementary Table 1. Reclassified variables**

| **Variable** | **Classification** | **Definition** |
| --- | --- | --- |
| Ethnicity | Indigenous | Aboriginal, Torres Strait Islander, Māori, Pacific peoples. |
|  | Any other ethnicity | - |
| Residence prior to hospital admission | Own home | - |
|  | Other residence | Sheltered living, residential care, nursing home or rehabilitation facility. |
| ASA score | <3 | - |
|  | ≥3 | - |
| Indication for surgery | Bleeding | Haemorrhage |
|  | Other | Abdominal wound dehiscence, abdominal compartment syndrome, planned relook, other |
|  | Obstruction | Small bowel obstruction, large bowel obstruction, hiatus hernia, incarcerated hernia, incisional hernia, internal hernia, intussusception, foreign body, pseudo-obstruction, volvulus |
|  | Sepsis | Phlegmon, pneumoperitoneum, sepsis, peritonitis, iatrogenic injury, anastomotic leak, bile leak, chyle leak, abdominal abscess, perforation |
|  | Ischaemia | Necrosis, ischemia, colitis, acidosis |
| Most senior surgeon in theatre | Consultant surgeon | Consultants, fellows, staff grade/other non-consultant grade responsible surgeon |
|  | Trainee surgeon | SET Training Resistrar, Serveice Registrar or equivalent |
| Most senior anesthetist in theatre | Consultant anesthetist | consultants, fellows, staff grade/other non-consultant grade responsible surgeon |
|  | Trainee anesthetist | Basic trainee, advanced trainee (pre- and post-final exam) or other trainees |

**Supplementary Table 2. Procedures**

| **Variable** | **Total**  **(n=8293)** | **Survivors**  **(n=7756)** | **Non-survivors**  **(n=537)** |
| --- | --- | --- | --- |
| Abscess- drainage of abscess/collection | 153 (1.9) | 146 (1.9) | 7 (1.3) |
| Abdominal wall closure following dehiscence | 30 (0.4) | 29 (0.4) | 1 (0.2) |
| Abdominal wall reconstruction | 37 (0.4) | 37 (0.5) | 0 (0.0) |
| Adhesiolysis | 1656 (20.0) | 1607 (20.7) | 49 (9.1) |
| Anastomosis-repair or revision of | 69 (0.8) | 64 (0.8) | 5 (0.9) |
| Appendicectomy as incidental | 17 (0.2) | 17 (0.2) | 0 (0.0) |
| Biliary reconstruction | 7 (0.1) | 7 (0.1) | 0 (0.0) |
| Cholecystectomy as incidental | 11 (0.1) | 9 (0.1) | 2 (0.4) |
| Left colectomy ^a^ | 408 (4.9) | 395 (5.1) | 13 (2.4) |
| Right colectomy ^b^ | 852 (10.3) | 806 (10.4) | 46 (8.6) |
| Subtotal colectomy ^c^ | 252 (3.0) | 222 (2.9) | 30 (5.6) |
| Hartmann’s procedure | 735 (8.9) | 692 (8.9) | 43 (8.0) |
| Other colorectal resection | 135 (1.6) | 119 (1.5) | 16 (3.0) |
| Debridement | 15 (0.2) | 13 (0.2) | 2 (0.4) |
| Enterotomy | 53 (0.6) | 51 (0.7) | 2 (0.4) |
| Foreign body removal | 60 (0.7) | 59 (0.7) | 1 (0.2) |
| Partial or total gastrectomy | 57 (0.7) | 50 (0.6) | 7 (1.3) |
| Gastric surgery | 40 (0.5) | 35 (0.5) | 5 (0.9) |
| Haematoma evacuation | 78 (0.9) | 71 (0.9) | 7 (1.3) |
| Haemostasis | 63 (0.8) | 55 (0.7) | 8 (1.5) |
| Hiatus hernia repair | 18 (0.2) | 18 (0.2) | 0 (0.0) |
| Intestinal bypass | 28 (0.3) | 26 (0.3) | 2 (0.4) |
| Intestinal fistula repair of | 9 (0.1) | 8 (0.1) | 1 (0.2) |
| Incisional hernia repair- large with bowel resection | 31 (0.4) | 29 (0.4) | 2 (0.4) |
| Incisional hernia repair- large with division of adhesions | 204 (2.5) | 200 (2.6) | 4 (0.7) |
| Exploratory/relook laparotomy | 427 (5.1) | 362 (4.7) | 65 (12.1) |
| Laparostomy formation | 34 (0.4) | 26 (0.3) | 8 (1.5) |
| Meckel’s diverticulum - resection | 45 (0.5) | 45 (0.6) | 0 (0.0) |
| Perforation - repair of intestinal perforation | 168 (2.0) | 158 (2.0) | 10 (1.9) |
| Peptic ulcer - suture or repair of perforation | 374 (4.5) | 350 (4.5) | 24 (4.5) |
| Peptic ulcer - oversew of bleed | 29 (0.3) | 26 (0.3) | 3 (0.6) |
| Tumour - resection of other intra-abdominal tumour(s) | 16 (0.2) | 15 (0.2) | 1 (0.2) |
| Small bowel resection | 1041 (12.6) | 969 (12.5) | 72 (13.4) |
| Stricturoplasty | 5 (0.1) | 5 (0.1) | 0 (0.0) |
| Stoma - Defunctioning stoma via midline laparotomy | 153 (1.8) | 142 (1.8) | 11 (2.0) |
| Stoma – Revision of stoma via midline laparotomy | 11 (0.1) | 11 (0.1) | 0 (0.0) |
| Volvulus - reduction | 51 (0.6) | 44 (0.6) | 7 (1.3) |
| Washout | 144 (1.7) | 133 (1.7) | 11 (2.0) |
| Other | 501 (6.0) | 461 (5.9) | 40 (7.4) |
| Not amendable to surgery | 27 (0.3) | 11 (0.1) | 16 (3.0) |
| Missing | 249 (3.0) | 233 (3.0) | 16 (3.0) |

Values are n (%) unless otherwise indicated.

^a^Left colectomy included sigmoid colectomy and anterior resection.

^b^Right colectomy included ileocaecal resection.

^c^Subtotal colectomy included panproctocolectomy.

**Supplementary Table 3. Baseline patient characteristics with missing data before multiple imputations**

| **Variable** | **Total**  **(n=8293)** | **Survivors**  **(n=7756)** | **Non-survivors**  **(n=537)** | **P value** | **Missingness (%)** |
| --- | --- | --- | --- | --- | --- |
| **Median age (IQR), years** | 66 (52-77) | 65 (52-76) | 74 (64-82) | <0.001 | 0.0 |
| **Sex** |  |  |  | 0.019 | 0.1 |
| Male | 4084 (49.2) | 3793 (48.9) | 291 (54.2) |  |  |
| Female | 4200 (50.8) | 3954 (51.1) | 246 (45.8) |  |  |
| **Ethnicity** |  |  |  | 0.294 | 0.0 |
| Indigenous | 1527 (18.4) | 1419 (18.3) | 108 (20.1) |  |  |
| Any other ethnicity | 6766 (81.6) | 6337 (81.7) | 429 (79.9) |  |  |
| **Admission type** |  |  |  | 0.159 | 0.4 |
| Elective | 446 (5.4) | 410 (5.3) | 36 (6.7) |  |  |
| Emergency | 7817 (94.3) | 7318 (94.4) | 499 (92.9) |  |  |
| **Readmission within 30 days for a previous EL** |  |  |  | 0.977 | 1.9 |
| Yes | 201 (2.4) | 188 (2.4) | 13 (2.4) |  |  |
| No | 7931 (95.6) | 7422 (95.7) | 509 (94.8) |  |  |
| **Admitted via ED** |  |  |  | 0.061 | 1.0 |
| Yes | 7286 (87.9) | 6827 (88.0) | 459 (85.5) |  |  |
| No | 923 (11.1) | 850 (11.0) | 73 (13.6) |  |  |
| **Residence prior to hospital admission** |  |  |  | <0.001 | 1.9 |
| Own home | 7723 (93.1) | 7244 (93.4) | 479 (89.2) |  |  |
| Other residence | 415 (5.0) | 366 (4.7) | 49 (9.1) |  |  |
| **Transfer from another hospital** |  |  |  | 0.042 | 2.3 |
| Yes | 1879 (22.7) | 1739 (22.4) | 140 (26.1) |  |  |
| No | 6226 (75.1) | 5844 (75.3) | 382 (71.1) |  |  |
| **Time from first arrival to seen by any member of surgical team** |  |  |  | 0.011 | 3.3 |
| <24 hours | 6120 (73.8) | 5747 (74.1) | 373 (69.5) |  |  |
| ≥24 hours | 1902 (22.9) | 1755 (22.6) | 147 (27.4) |  |  |
| **Time from first arrival to knife to skin** |  |  |  | 0.886 | 9.2 |
| < 24 hours | 2644 (31.9) | 2475 (31.9) | 169 (31.5) |  |  |
| ≥ 24 hours | 4883 (58.9) | 4575 (59.0) | 308 (57.4) |  |  |
| **Time from first arrival to administration of antibiotics** |  |  |  | 0.704 | 22.0 |
| < 24 hours | 3816 (46.0) | 3567 (46.0) | 249 (46.4) |  |  |
| ≥ 24 hours | 2655 (32.0) | 2488 (32.1) | 167 (31.1) |  |  |
| **Preoperative diagnostic abdominal CT scan performed** |  |  |  | <0.001 | 1.9 |
| Yes | 7527 (90.8) | 7058 (91.0) | 469 (87.3) |  |  |
| No | 607 (7.3) | 547 (7.1) | 60 (11.2) |  |  |
| **Suspision of sepsis at the time of initial admission** |  |  |  | <0.001 | 5.6 |
| Yes | 2174 (26.2) | 1994 (25.7) | 180 (33.5) |  |  |
| No | 5658 (68.2) | 5340 (68.8) | 318 (59.2) |  |  |
| **Suspision of sepsis at the time of decision for surgery** |  |  |  | <0.001 | 7.2 |
| Yes | 2651 (32.0) | 2387 (30.8) | 264 (49.2) |  |  |
| No | 5044 (60.8) | 4809 (62.0) | 235 (43.8) |  |  |
| **Lactate level (mmol/L), median (IQR)** | 1.6 (1.1-2.4) | 1.5 (1.1-2.3) | 2.6 (1.5-4.8) | <0.001 | 28.9 |
| **ASA score** |  |  |  | <0.001 | 25.7 |
| <3 | 2228 (26.9) | 2199 (28.4) | 29 (5.4) |  |  |
| ≥3 | 3932 (47.4) | 3575 (46.1) | 357 (66.5) |  |  |
| **Urgency (hours), Median (IQR)** | 6 (4-12) | 6 (4-12) | 4 (2-6) | <0.001 | 0.0 |
| **First procedure within this admission** |  |  |  | <0.001 | 0.0 |
| Yes | 7361 (88.8) | 6925 (89.3) | 436 (81.2) |  |  |
| No | 932 (11.2) | 831 (10.7) | 101 (18.8) |  |  |
| **Indication for surgery**^a^ |  |  |  |  | 0.0 |
| Bleeding | 412 (5.0) | 374 (4.8) | 38 (7.1) | 0.020 |  |
| Obstruction | 4534 (54.7) | 4342 (56.0) | 192 (35.8) | <0.001 |  |
| Sepsis | 3008 (36.3) | 2794 (36.0) | 214 (39.9) | 0.074 |  |
| Ischaemia | 897 (10.8) | 753 (9.7) | 144 (26.8) | <0.001 |  |
| Other | 107 (1.3) | 87 (1.1) | 20 (3.7) | <0.001 |  |
| **Most senior surgeon in theatre** |  |  |  | 0.003 | 2.1 |
| Consultant surgeon | 8019 (96.7) | 7494 (96.6) | 525 (97.8) |  |  |
| Trainee surgeon | 96 (1.2) | 96 (100.0) | 0 (0.0) |  |  |
| **Most senior anesthetist in theatre** |  |  |  | 0.066 | 5.1 |
| Consultant anesthetist | 7382 (89.0) | 6893 (88.9) | 489 (91.1) |  |  |
| Trainee anesthetist | 488 (5.9) | 466 (6.0) | 22 (4.1) |  |  |

Values are n (%) unless otherwise indicated.

ASU, acute surgical unit; CT, computed tomography; NELA, national emergency laparotomy audit; ASA, American Society of Anaesthesiologists; EL, emergency laparotomy.

^a^ Patients can have more than one indication for surgery.

**Supplementary Table 4. Baseline patient characteristics and primary outcome**

| **Variable** | **Training (n=6619)** | **Testing (n=1674)** | **P value** |
| --- | --- | --- | --- |
| **Median age (IQR), years** | 66 (52-77) | 66 (52-77) | 0.830 |
| **Sex** |  |  | 0.050 |
| Male | 3301 (49.9) | 790 (47.2) |  |
| Female | 3318 (50.1) | 884 (52.8) |  |
| **Ethnicity** |  |  | 0.019 |
| Indigenous | 1252 (18.9) | 275 (16.4) |  |
| Any other ethnicity | 5367 (81.1) | 1399 (83.6) |  |
| **Admission type** |  |  | 0.485 |
| Elective | 351 (5.3) | 96 (5.7) |  |
| Emergency | 6268 (94.7) | 1578 (94.3) |  |
| **Readmission within 30 days for a previous EL** |  |  | 0.666 |
| Yes | 158 (2.4) | 43 (2.6) |  |
| No | 6461 (97.6) | 1631 (97.4) |  |
| **Admitted via ED** |  |  | 0.240 |
| Yes | 5868 (88.7) | 1501 (89.7) |  |
| No | 751 (11.3) | 173 (10.3) |  |
| **Residence prior to hospital admission** |  |  | 0.636 |
| Own home | 6284 (94.9) | 1594 (95.2) |  |
| Other residence | 335 (5.1) | 80 (4.8) |  |
| **Transfer from another hospital** |  |  | 0.674 |
| Yes | 1514 (22.9) | 391 (23.4) |  |
| No | 5105 (77.1) | 1283 (76.6) |  |
| **Time from first arrival to seen by any member of surgical team** |  |  | 0.171 |
| <24 hours | 1572 (23.7) | 371 (22.2) |  |
| ≥24 hours | 5047 (76.3) | 1303 (77.8) |  |
| **Time from first arrival to knife to skin** |  |  | 0.304 |
| < 24 hours | 4296 (64.9) | 1064 (63.6) |  |
| ≥ 24 hours | 2323 (35.1) | 610 (36.4) |  |
| **Time from first arrival to administration of antibiotics** |  |  | 0.149 |
| < 24 hours | 2929 (44.3) | 708 (42.3) |  |
| ≥ 24 hours | 3690 (55.7) | 966 (57.7) |  |
| **Preoperative diagnostic abdominal CT scan performed** |  |  | 0.727 |
| Yes | 6128 (92.6) | 1554 (92.8) |  |
| No | 491 (7.4) | 120 (7.2) |  |
| **Suspision of sepsis at the time of initial admission** |  |  | 0.571 |
| Yes | 1841 (27.8) | 454 (27.1) |  |
| No | 4778 (72.2) | 1220 (72.9) |  |
| **Suspision of sepsis at the time of decision for surgery** |  |  | 0.438 |
| Yes | 2265 (34.2) | 556 (33.2) |  |
| No | 4354 (65.8) | 1118 (66.8) |  |
| **Lactate level (mmol/L), median (IQR)** | 1.9 (1.3-2.7) | 1.8 (1.2-2.7) | 0.527 |
| **ASA score** |  |  | 0.606 |
| <3 | 4484 (67.7) | 1123 (67.1) |  |
| ≥3 | 2135 (32.3) | 551 (32.9) |  |
| **Urgency (hours), median (IQR)** | 6 (4-12) | 6 (4-12) | 0.895 |
| **First procedure within this admission** |  |  | 0.657 |
| Yes | 5870 (88.7) | 1491 (89.1) |  |
| No | 749 (11.3) | 183 (10.9) |  |
| **Indication for surgery**^a^ |  |  |  |
| Bleeding | 321 (4.8) | 91 (5.4) | 0.324 |
| Obstruction | 3632 (54.9) | 902 (53.9) | 0.468 |
| Sepsis | 2398 (36.2) | 610 (36.4) | 0.873 |
| Ischaemia | 712 (10.8) | 185 (11.1) | 0.729 |
| Other | 91 (1.4) | 16 (1.0) | 0.175 |
| **Most senior surgeon in theatre** |  |  | 0.874 |
| Consultant surgeon | 6543 (98.9) | 1654 (98.8) |  |
| Trainee surgeon | 76 (1.1) | 20 (1.2) |  |
| **Most senior anesthetist in theatre** |  |  | 0.347 |
| Consultant anesthetist | 6219 (94.0) | 1583 (94.6) |  |
| Trainee anesthetist | 400 (6.0) | 91 (5.4) |  |
| **Survival status** |  |  | 0.411 |
| Survivors | 436 (6.6) | 101 (6.0) |  |
| Non-survivors | 6183 (93.4 ) | 1573 (94.0) |  |

Values are n (%) unless otherwise indicated.

**Supplementary Table 5. Multivariate logistic regression**

| **Variables** | **β coefficient** | **Wald statistics** | **OR** | **P value** |
| --- | --- | --- | --- | --- |
| **Age (years)** | 0.04 | 92.84 | 1.04 (1.03, 1.05) | **<0.001** |
| **Sex** |  |  |  |  |
| Female | Reference | Reference | Reference |  |
| Male | 0.15 | 2.08 | 1.17 (0.95, 1.44) | 0.149 |
| **Residence prior to hospital admission** |  |  |  |  |
| Other | Reference | Reference | Reference |  |
| Own home | -0.30 | 2.29 | 0.74 (0.51, 1.09) | 0.130 |
| **Transfer from another hospital** |  |  |  |  |
| No | Reference | Reference | Reference |  |
| Yes | 0.13 | 1.11 | 1.14 (0.90, 1.44) | 0.293 |
| **Time from first arrival to first seen by any member of surgical team** |  |  |  |  |
| < 24 hours | Reference | Reference | Reference |  |
| ≥ 24 hours | 0.17 | 2.12 | 1.19 (0.94, 1.50) | 0.145 |
| **Preoperative diagnostic abdominal CT scan performed** |  |  |  |  |
| No | Reference | Reference | Reference |  |
| Yes | -0.51 | 7.74 | 0.60 (0.42, 0.86) | **0.005** |
| **Suspicion of sepsis at the time of initial admission** |  |  |  |  |
| No | Reference | Reference | Reference |  |
| Yes | -0.25 | 2.48 | 0.78 (0.57, 1.06) | 0.115 |
| **Suspicion of sepsis at the time of decision for surgery** |  |  |  |  |
| No | Reference | Reference | Reference |  |
| Yes | 0.60 | 14.60 | 1.83 (1.34, 2.50) | **<0.001** |
| **Lactate (mmol/l)** | 0.19 | 83.63 | 1.21 (1.16, 1.26) | **<0.001** |
| **ASA score** |  |  |  |  |
| <3 | Reference | Reference | Reference |  |
| ≥3 | 1.28 | 43.44 | 3.58 (2.45, 5.23) | **<0.001** |
| **Urgency (hours)** | -0.03 | 10.22 | 0.98 (0.96, 0.99) | **0.001** |
| **First procedure within this admission** |  |  |  |  |
| No | Reference | Reference | Reference |  |
| Yes | -0.26 | 2.87 | 0.77 (0.57, 1.04) | 0.09 |
| **Indication for surgery - Bleeding** |  |  |  |  |
| No | Reference | Reference | Reference |  |
| Yes | -0.03 | 0.02 | 0.97 (0.60, 1.56) | 0.895 |
| **Indication for surgery - Obstruction** |  |  |  |  |
| No | Reference | Reference | Reference |  |
| Yes | -0.42 | 9.88 | 0.66 (0.51, 0.86) | **0.002** |
| **Indication for surgery - Ischaemia** |  |  |  |  |
| No | Reference | Reference | Reference |  |
| Yes | 0.78 | 35.49 | 2.18 (1.69, 2.81) | **<0.001** |
| **Indication for surgery - Other** |  |  |  |  |
| No | Reference | Reference | Reference |  |
| Yes | 0.88 | 7.91 | 2.41 (1.31, 4.44) | **0.005** |
| **Most senior surgeon in theatre** ^a^ | n/a | n/a | n/a | n/a |

Values in parentheses are 95% confidence intervals.

n/a, not applicable; CT, computed tomography; ASA, American Society of Anesthesiologists; OR, odds ratio.

^a^This parameter is redundant and thus was discarded from the final multivariate logistic regression model.

**Supplementary Table 6. Variable importance**

|  | **Importance (%)** | | | |
| --- | --- | --- | --- | --- |
|  | **MLP extended** | **RBF extended** | **MLP simplified** | **RBF simplified** |
| Age | 61.3 | 62.1 | 97.4 | 83.4 |
| Sex | 4.9 | 4.9 | 9.4 | 9.2 |
| Ethnicity | 6.3 | 10.7 | n/a | n/a |
| Admission type | 5.7 | 18.1 | n/a | n/a |
| Readmission within 30 days for a previous EL | 6.1 | 14.9 | n/a | n/a |
| Admitted via ED | 5.0 | 17.1 | n/a | n/a |
| Residence prior to hospital admission | 22.0 | 15.5 | 2.4 | 27.2 |
| Transfer from another hospital | 3.1 | 9.2 | 12.3 | 29.0 |
| Time from first arrival to seen by any member of surgical team | 5.3 | 18.8 | 10.7 | 29.6 |
| Time from first arrival to knife to skin | 4.4 | 19.2 | n/a | n/a |
| Time from first arrival to administration of antibiotics | 4.4 | 17.1 | n/a | n/a |
| Preoperative diagnostic abdominal CT scan performed | 4.1 | 15.5 | 20.0 | 29.7 |
| Suspision of sepsis at the time of initial admission | 4.1 | 46.5 | 7.3 | 54.3 |
| Suspision of sepsis at the time of decision for surgery | 11.2 | 48.8 | 9.4 | 69.5 |
| Lactate (mmol/L) | 100.0 | 100.0 | 100.0 | 75.8 |
| ASA score | 21.2 | 13.3 | 35.9 | 21.6 |
| Urgency (hours) | 9.0 | 37.7 | 21.7 | 74.4 |
| First procedure within this admission | 10.9 | 23.5 | 15.4 | 30.8 |
| Surgical Indication- Bleeding | 9.1 | 20.0 | 2.8 | 29.4 |
| Surgical Indication- Obstruction | 5.1 | 30.8 | 7.5 | 41.1 |
| Surgical Indication- Sepsis | 5.7 | 34.6 | n/a | n/a |
| Surgical Indication- Ischaemia | 12.4 | 36.5 | 25.8 | 100.0 |
| Surgical Indication- Other | 14.7 | 17.2 | 13.7 | 24.6 |
| Most senior surgeon in theatre | 9.4 | 15.0 | 8.5 | 24.0 |
| Most senior anesthetist in theatre | 8.7 | 13.3 | n/a | n/a |

n/a, not applicable; CT, computed tomography; ASA, American Society of Anesthesiologists; OR, odds ratio.

**Supplementary Table 7 Sensitivity analysis assessing model discrimination using complete-case analysis without multiple imputation**

| **Models** | **Original analysis (MICE-imputed AUROC (95% CI)** | **Complete-case analysis AUROC (95% CI)** |
| --- | --- | --- |
| **Training** | **n=6619** | **n=2356** |
| NELA | 0.832 (0.815, 0.850) | 0.841 (0.814, 0.869) |
| MLP simplified | 0.810 (0.791, 0.829) | 0.854 (0.829, 0.879) |
| Multivariate LR | 0.806 (0.788, 0.825) | 0.793 (0.762, 0.824) |
| MLP extended | 0.792 (0.772, 0.812) | 0.819 (0.790, 0.848) |
| RBF extended | 0.738 (0.715, 0.761) | 0.598 (0.556-0.641) |
| RBF simplified | 0.698 (0.673, 0.723) | 0.646 (0.604, 0.689) |
| **Testing** | **n=1674** | **n=588** |
| NELA | 0.836 (0.799, 0.872) | 0.828 (0.776, 0.880) |
| Multivariate LR | 0.824 (0.790, 0.858) | 0.762 (0.693, 0.830) |
| MLP simplified | 0.817 (0.781, 0.854) | 0.727 (0.650, 0.803) |
| MLP extended | 0.802 (0.762, 0.843) | 0.722 (0.643, 0.800) |
| RBF extended | 0.719 (0.674, 0.764) | 0.646 (0.554, 0.739) |
| RBF simplified | 0.672 (0.620, 0.724) | 0.672 (0.584, 0.761) |

MICE, multiple imputation by chained equations; CI, confidence interval; AUROC, area under the receiver operating characteristics curve; RBF, radial basis function; MLP, multilayer perceptron; LR, logistic regression; NELA, national emergency laparotomy audit risk calculator

**Table S8 Calibration performance in the testing dataset**

| **Model** | **Calibration intercept (95% CI)** | **Calibration slope (95% CI)** | **Brier score** |
| --- | --- | --- | --- |
| **NELA** | -0.616 (-0.979, -0.254) | 1.014 (0.819, 1.209) | 0.053 |
| **MLP simplified** | -0.400 (-0.797, -0.004) | 0.862 (0.700, 1.023) | 0.051 |
| **MLP extended** | -0.529 (-0.929, -0.130) | 0.752 (0.611, 0.893) | 0.053 |
| **RBF simplified** | -1.196 (-1.986, -0.405) | 0.559 (0.264, 0.854) | 0.056 |
| **RBF extended** | -1.008 (-1.752, -0.264) | 0.620 (0.330, 0.909) | 0.055 |
| **Multivariate LR** | -0.104 (-0.567, 0.359) | 1.005 (0.811, 1.200) | 0.051 |

CI, confidence interval; RBF, radial basis function; MLP, multilayer perceptron; LR, logistic regression; NELA, national emergency laparotomy audit risk calculator
